# Environmental Exposure to the Common Trunk of Mammalian Appeasing Pheromone Modulates Social Behavior and Reduces Fight Wounds in Male Mice

**DOI:** 10.3390/ani15223278

**Published:** 2025-11-13

**Authors:** Sara Fuochi, Cecile Bienboire-Frosini, Estelle Descout, Miriam Marcet-Rius, Patrick Pageat, Alessandro Cozzi

**Affiliations:** 1 Department of Ethics, Legislation & Animal Welfare, Research Institute in Semiochemistry and Applied Ethology (IRSEA), 84400 Apt, France; 2 Department of Molecular Biology and Chemical Communication, Research Institute in Semiochemistry and Applied Ethology (IRSEA), 84400 Apt, France; 3 Statistics and Data Management Service, Research Institute in Semiochemistry and Applied Ethology (ISEA), 84400 Apt, France; 4 Research and Education Board, Research Institute in Semiochemistry and Applied Ethology (IRSEA), 84400 Apt, France

**Keywords:** laboratory mouse, prosocial behaviors, aggression, fight wounds, mammalian appeasing pheromone

## Abstract

Aggression among group-housed male laboratory mice poses significant challenges for animal welfare and scientific outcomes. Semiochemicals, such as mammalian appeasing pheromones, have shown potential in modulating social behaviors in various species. This study aimed to evaluate the effects of a synthetic Common Trunk (CT) of mammalian appeasing pheromone on social behavior, aggression, and welfare indicators in adult male mice. Specific Pathogen Free (SPF) male RjOrl:SWISS mice (8–12 weeks old) were housed in open-top cages and exposed via environmental passive diffusion to either a 2% synthetic CT pheromone formulation or a placebo (excipient only). Behavioral tests included the Elevated Plus Maze (EPM) and the Resident–Intruder (RI) test. Clinical welfare assessments and hematobiochemical analyses were also performed. Behavioral testing revealed minimal differences between groups, except for—in animals exposed to the CT—significantly fewer unsupported rearings, both in number (*p* = 0.0284) and duration (*p* = 0.0184), suggesting reduced vigilance (EPM Test) and shorter upright posture durations (*p* = 0.0031), a behavior linked to social signaling during conflict (RI test). Welfare assessments and risk-based analyses indicated that mice exposed to the CT of the appeasing pheromone displayed more agonistic but less violent confrontations, with fewer visible lesions. The protective effect was most pronounced during early group housing of unfamiliar adult males, with significantly fewer injuries in treated mice during the first (*p* = 0.0215) and second week (*p* = 0.0329). Treated mice also showed higher serotonin levels (*p* = 0.0295), suggesting reduced aggressiveness in line with observed behavioral outcomes. Exposure to the CT appeared to improve social dynamics and reduce escalation of aggression in male mice, supporting its potential as a refinement tool in laboratory housing practices.

## Simple Summary

Aggression among male laboratory mice housed together is a common problem in research facilities, often leading to injuries, stress, and the need to separate or cull animals, which negatively impacts their welfare and the reliability of scientific results. This study investigated whether exposure to a synthetic version of a chemical signal, called mammalian appeasing pheromone, could reduce aggression and modulate social behavior in male mice. We assessed a range of behavioral tests, injury rates, and blood parameters related to stress and aggression. While behavioral tests showed only limited differences, animals treated with the pheromone displayed clearer signs of improved welfare: they had fewer injuries, engaged more in non-violent communication behaviors, and showed changes in serotonin levels associated with aggression control. The protective effects were most evident during the early stages of group housing. Our findings suggest that this non-invasive treatment could reduce aggressive behavior in group-housed male mice, improving animal welfare in laboratory settings and potentially shaping better housing strategies in both research and breeding environments.

## 1. Introduction

Social interactions are central to the health and psychological well-being of social animals [1]. Prosocial behaviors are defined as actions that an individual performs to benefit others, which are expected to produce or maintain the physical and psychological well-being of others [2]. They could be positive welfare indicators of a bilateral nature, beneficial to the recipient and rewarding to the donor, as they elicit positive emotions [3]. Previous studies conducted by various research teams have demonstrated that the application of mammalian appeasing pheromone could have beneficial effects on both animals and humans [4,5,6,7,8]. Whether the same application and approach could benefit laboratory animals, especially rodents, is a brand-new line of research, aiming to further boost refinement [9]. According to the 2022 European Commission Report on the number of animals used in research covering EU27 and Norway [10], the mouse is still the most used experimental animal, providing 47.83% of all animals used, accounting for the remarkable number of 4,010,766 individuals. Common issues in this species in the laboratory setting include aggressivity, fight wounds, and the inability to pool together adult unfamiliar male mice, leading to single-housing or suboptimal use of cage contingency [11,12,13]. Consequently, both breeding and experimental facilities, as well as millions of mice, could potentially benefit from the application of a suitable appeasing pheromone, as demonstrated with other species. Unlike other species, though, a mouse-specific appeasing pheromone has not yet been identified, characterized, or synthesized, and its effects remain unknown. Nevertheless, mammalian appeasing pheromones share a conserved biochemical core—referred to as the Common Trunk—mostly composed of oleic, palmitic, and linoleic acids, which is suggested to possess intrinsic appeasing properties across mammalian species, with species-specific fatty acid enhancers further potentiating this core signal [14,15]. Based on this rationale, we explored the hypothesis that the Common Trunk (CT) alone could exhibit appeasing efficacy in male mice, despite lacking mouse-specific enhancers, through behavioral testing, social and welfare assessments, and hematobiochemical stress indicators.

## 2. Materials and Methods

### 2.1. Animals

SPF (Specific Pathogen Free) male mice in the range of 8–12 weeks-old were purchased from Janvier Labs, le Genest Saint Isle, France (strain RjOrl:SWISS, outbred). Identification of each animal was achieved by subcutaneous microchip implantation at the Vendor site before shipment. At IRSEA, Apt, France, animals were housed in open cages (1284L Eurostandard Type II L Tecniplast, Buguggiate, Italy) at a standardized density of five mice/cage, provided with ad libitum water and a standard rodent maintenance diet (Mucedola Global Diet 2016, distributed by Inotiv, West Lafayette, IN, USA). Cage changes were performed weekly using dust-free wooden bedding (Scobis Quattro, Mucedola, Settimo Milanese, Italy). Cages were enriched with a variety of environmental enrichment items, both disposable and reusable, including disposable cardboard mouse igloos (SmartHome, Datesand, Stockport, UK), tunnels (Tecniplast), nesting materials such as paper SizzleNest (Datesand), chewing aspen blocks (Tapvei, Harjumaa, Estonia), and cotton cocoons (Datesand). Tunnel handling was the standard method applied to move or handle the mice, also for experimental procedures—e.g., placement on the maze; transfer of an intruder into the resident cage. A partially inverted 12:12 light cycle was set to have lights on in the interval 00 h 30–12 h 30 and lights off in the interval 12 h 30–00 h 30. Red lights were used in the behavioral testing areas during the dark period. Dedicated personnel and technicians were assigned to the respective rooms, with workflows and gowning procedures adapted to prevent cross-contamination between the two groups. Cages were assigned proportionally to Room A (Group A, Placebo) and Room B (Group B, Common Trunk) upon arrival at the Institute, following the criteria of age matching across groups. All animals were sourced in a single delivery batch from the same supplier, where mice were bred under vendor genetic policy, and exhibited comparable characteristics upon arrival. This allocation strategy was designed to minimize baseline differences between groups while maintaining consistent housing and handling conditions throughout the study. The experimental unit was the room, as treatment and placebo were administered at the room level via environmental diffusion. In each room and group, there were 50 animals, consisting of 25 two-month-old mice at arrival, referred to as “intruders,” and 25 three-month-old mice at arrival, referred to as “residents”. Twelve supplementary animals (six per room, three intruders and three residents) were made available for preliminary assessments of test settings or as spare individuals in case of issues in the acclimation month, and were housed at a density of three mice/cage. A total of 112 animals were involved in the different phases of this study. The group size of 50 animals was determined a priori. Given the exploratory nature and complexity of our study design, including group housing, environmental diffusion of the treatment, and social interaction tests, we adopted a pragmatic and ethically conscious approach. This sample size reflects a compromise between minimizing animal use and yet ensuring an acceptable statistical power of 70%, with α = 0.05.

### 2.2. Treatment, Groups, and Timeline

**Figure 1:**
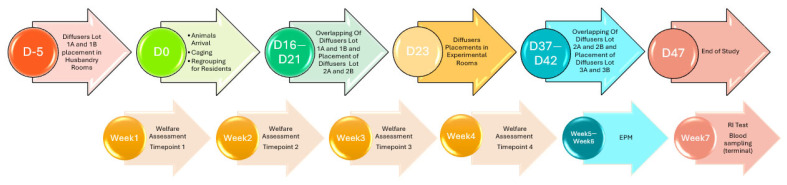
Study timeline, including duration, testing windows, and placements of diffusers. Groups A and B refer to treatment groups, with A being Placebo and B being CT. Progressive numbers (1, 2, and 3) indicate subsequent batches placed over time. EPM indicates Elevated Plus Maze and RI indicates Resident–Intruder Test.

The treatment consisted of a 2% formulation of synthetic Common Trunk (CT) of mammalian appeasing pheromone [15], with 3-methoxy-3-methyl-1-butanol serving as the excipient. In contrast, the placebo was composed solely of this excipient. Both the treatment and the placebo were administered through an environmental passive capillary diffusion system (PCDS) [6], exposing the animals to either the treatment or the placebo (see Figure 1). Both formulations were produced by SIGNS Labs, Apt, France.

The tests followed a double-blind procedure to maintain impartiality. Only the personnel from SIGNS Labs, who manufactured the product, knew the correspondence between the groups A and B and the nature of the treatments (CT of pheromones or placebo). The rest of the team was unaware of this information during the study. Both the analysts and statisticians analyzed the data without knowing which product was used, referring to them as A and B. Treatment details were only revealed after the statistical analyses, with Group A being the Placebo and Group B the Verum (CT of pheromones).

The rooms’ conditioning, animal habituation, and experimental timeline covered two months (Figure 1). The process began several days before the animals’ arrival with the preparation of the husbandry and experimental rooms. On Day-5, diffusers were placed in Rooms A and B, using Lots 1A and 1B to establish the environmental conditions before the animals were introduced. The animals arrived on Day 0, were caged, and assigned to either Room A or Room B, marking the start of the study. The conditioning of the rooms involved a progressive replacement of diffuser lots. Between Days 16 and 21, the diffusers from Lots 1A and 1B remained in place, while new diffusers from Lots 2A and 2B were introduced. This overlap ensured continuous room saturation with either the treatment or placebo. From Days 37 to 42, the diffusers from Lots 2A and 2B remained active while new diffusers from Lots 3A and 3B were introduced, further ensuring proper room saturation until the study’s completion. The experimental phases were carried out over multiple following weeks. The first four weeks were dedicated to welfare assessments, focusing on group dynamics in pre-existing and newly mixed groups. This also allowed for acclimatization before subsequent behavioral tests. Between Weeks 5 and 6, the Elevated Plus Maze (EPM) test was performed, while in Week 7, the Resident–Intruder (RI) test was conducted, followed by terminal blood sampling for further physiological data collection. The study concluded on Day 47, with all data collected covering welfare, behavior (EPM and RI), and blood sampling for physiological measurements.

### 2.3. Video Analysis

The entire duration of all tests was filmed with Sony Handycam HDR-CX625 cameras (Sony, Tokyo, Japan) and analyzed with Behavioral Observation Research Interactive Software (BORIS version 8.20, 1 June 2023). Videos from the behaviors related to EPM and RI tests were examined by two different operators.

### 2.4. Welfare Assessment

The assessment consisted of multiple observations of each animal, and an assessment of each in the groups (cage). This assessment covered all the animals received, including spare ones (*n* = 112), and was performed by trained personnel, blinded to treatment, once a week during the first month at the Institute (acclimation phase before behavioral tests). Parameters assessed fell into the domains of physical conditions, behavior, group dynamics, and human–mouse interaction (approach to human hand in the cage). Full details are available in the Supplementary Material, Supplementary File S1 and Supplementary Table S1.

### 2.5. Elevated Plus Maze (EPM) Test

The EPM test was performed on 100 individual mice over a 2-week period at a rate of 10 mice tested per day (5 mice/group, so one cage/group). A total of three animals were removed from analysis due to misplacement/falling from the maze (*n* = 2, one from Group A and one from Group B) and malfunctioning of the camera (*n* = 1, from Group B). The EPM test is a widely established and extensively published method for assessing anxiety and the effects of anxiety-reducing treatments, as well as evaluating behavioral changes in response to stress [16,17,18,19,20,21,22]. Given the exploratory and first-of-its-kind nature of this study, testing mice treated with mammalian appeasing pheromones in the EPM allowed us, on the one hand, to use a standardized analytical approach and, on the other hand, to assess its relevance and applicability to our model. The parameters considered were adapted to our scope from the existing literature [16,17,18,19,20,21,22] and are summarized in Table 1.

### 2.6. Resident–Intruder (RI) Test

The RI test involved 96 individual mice over a 1-week period at an average rate of 20 mice per day: 5 intruder mice and 5 resident mice per group/room. Data from a total of 48 residents were analyzed (*n* = 23 from Group A, and *n* = 25 from Group B). Two residents from Group A were removed from the experimental cohort due to poor clinical conditions (exclusion criteria). The mice were tested in the same order as they were tested in the EPM test during the previous two weeks. The resident was isolated in single housing for one week, before the day of the test. For this purpose, and accounting for extra space allowed for flight during the RI test, 1290D Eurostandard Type III Tecniplast cages were used. At the end of the test, the resident was euthanized for the blood sampling terminal procedure and the intruder returned to its cage. The total duration of each test was 5 min, unless blood traces were observed (endpoint criteria). The RI test was chosen due to its strong standardization [23], ability to assess the effects of treatments in modulating stress responses [24], and its capacity to quantify aggression and social stress [25], providing a solid foundation for our model given the exploratory and first-of-its-kind nature of our analysis. The parameters considered were adapted to our scope from the existing literature [23,24,25,26,27,28,29] and are summarized in Table 2.

### 2.7. Hematobiochemical Analysis

Blood was collected as a terminal procedure under anesthesia (Isoflurane) through a cardiac puncture from residents at the end of the Resident–Intruder test (*n* = 48). Blood was stored in heparin lithium vials (Sarstedt, Nümbrecht, Germany, #15.1673). Plasma was separated by centrifugation at 1500× *g* for 10 min and stored at −20 °C until analysis. Plasmatic testosterone, serotonin, and corticosterone concentrations were measured using commercial competitive enzyme-linked immunosorbent assays (Cayman Chemical, Ann Arbor, MI, USA, #582751 and Enzo Life Sciences, Farmingdale, NY, USA, #ADI-900-175, #ADI-900-097) following manufacturers’ instructions. Accordingly, the steroid displacement reagent was used to free steroids bound to plasma proteins when measuring corticosterone concentrations.

### 2.8. Inclusion, Exclusion and Endpoint Criteria

For this study, inclusion, non-inclusion, and exclusion criteria were defined a priori.

Inclusion criteria: animals of the defined age, sex, and strain, capable of expressing the full range of species-specific behaviors and without evident organic problems.

Non-inclusion criteria: animals posing a risk to personnel (based on clinical records and investigator judgment); those with sensory deficits (e.g., vision impairment); altered state of consciousness; neurological disorders; or organic disease.

Exclusion criteria: animals developing illness during the study; showing signs of extreme stress or emotional distress during testing; animals failing the behavioral tests because of a fall (EPM).

Details of any animals excluded from the experimental cohorts are reported separately for each analysis, when applicable. Physical injuries or severe fight wounds, also unrelated to EPM and RI tests, were identified as a humane endpoint. No other humane endpoints were considered critical due to the nature of the study. Although no others predefined humane endpoints were established as such, animals were under continuous clinical monitoring by experienced veterinary staff. Required interventions, if any, were based on individual clinical conditions independently from experimental procedures.

### 2.9. Statistical Analysis

Data analysis was carried out using R version 4.1.2 (1 November 2021). The significance threshold was fixed at 5%.

Prior to statistical analysis of behavioral parameters related to EPM and RI tests, inter-observer reliability was assessed. The Pearson coefficient was used when the normality condition was verified for each observer (thanks to the normality test and QQ-plot graphic); otherwise, the Spearman coefficient was chosen. The association between the two readers was computed by squaring the correlation coefficient obtained. An association ≥80% was required for analysis of the corresponding parameters. If the association was >80%, the mean of the two video readers was computed and used for the rest of the analysis. Otherwise, the variable was re-evaluated by the readers, or data from a single reader was retained.

For analysis, the statistical unit was defined according to the type of measurement: individual mice for biochemistry and the EPM behavioral test, pairs of mice for the RI test, and cages for well-being indicators.

For biochemical parameters, the effect of treatment (Placebo and CT) was tested. Corticosterone was evaluated thanks to a Student’s *t*-test. Conditions of normality (normality tests and graphically) and homogeneity of variances (using Fisher test) were checked. For Serotonin and Testosterone, a Wilcoxon test was employed due to the absence of normality.

For behavioral parameters related to EPM and RI tests, different statistical methods were used, depending on the type of each parameter. The effect of treatment (Placebo and CT) was evaluated as a fixed effect thanks to mixed models. Cage and Day were considered as random effects for the EPM test, and only Day for the RI test. For behaviors expressed as durations, General Linear Mixed Models (GLMMs) were applied. Conditions of residues normality (graphically and using normality tests) and homoscedasticity (“Residuals versus fits” graphic and Levene’s test) were verified. When these assumptions were not checked, the Box–Cox transformation was applied to the data. For several parameters, many zeros were detected, which implied the use of a Hurdle mixed model. For behaviors expressed as the number of occurrences, Generalized Linear Mixed Models (GzLMMs) with the Poisson distribution were performed as a first choice. The Pearson chi-square/DF statistic was used to evaluate the dispersion of the data. If this statistic was less than 2, no overdispersion was detected and the Poisson model was adapted. If it was higher than 2, the data were overdispersed and the Poisson model was not adequate. In that case, a negative binomial model was preferred. For behaviors expressed as latencies, a Cox frailty model for survival data was performed. The condition of proportional hazard was verified with the study of Schoenfeld residues. For behaviors expressed in binary, Generalized Linear Mixed Models (GzLMMs) were used with the Binomial distribution.

For welfare indicators, Generalized Linear Mixed Models (GzLMMs) were carried out to evaluate the effects of treatment (Placebo and CT), week (1–4), age (2 and 3 months), and their interactions (treatment x week and treatment x age) as fixed effects. Cage was considered as a random effect. For discrete variables, the Poisson distribution was performed as a first choice. The Pearson chi-square/DF statistic was used in order to evaluate the dispersion of the data. This statistic was less than 0.5 for two variables, which means that the data were underdispersed and the Poisson model was not adequate. In that case, a GzLMM was performed by analyzing the parameter according to the number of mice in each cage and by specifying the Binomial distribution. Finally, several parameters were binary coded, due to small variation in values (number of mice realizing the behavior), with only two modalities analyzed: 0 (no) and 1 (yes). For these parameters, GzLMMs were used with the Binomial distribution (binary response). Multiple comparisons were performed thanks to the Tukey test for effects with more than two modalities. Complete models were simplified if the AIC and BIC criteria decreased when an effect was removed from the model (and if this one was not significant). An additional analysis was performed to assess the effect of treatment, week by week, on the number of lesions, keeping only 3-month-old mice (unfamiliar regrouped animals). Poisson regression was performed. The Pearson chi-square/DF statistic was used in order to evaluate the dispersion of the data. These were not overdispersed, which made it possible to apply this type of model. Finally, the risk of injuries according to treatment was assessed over the four grouped weeks and on a week-by-week basis. For this, various key epidemiological indicators were calculated: injury incidence in each group, absolute risk reduction (ARR), and relative risk (RR). These measures were used to quantify the effect of the treatments on injury risk and to compare their respective effectiveness.

## 3. Results

Given the extensive number of parameters analyzed, the Results section focuses on statistically significant findings and other outcomes of relevance. All results, including non-significant ones, are provided in the Supplementary Material for completeness and transparency. Inter-observer association in the EPM and RI tests was lower than 80%; thus, below-threshold variables were reworked by the readers to find consensus.

### 3.1. Welfare Assessment

**Figure 2:**
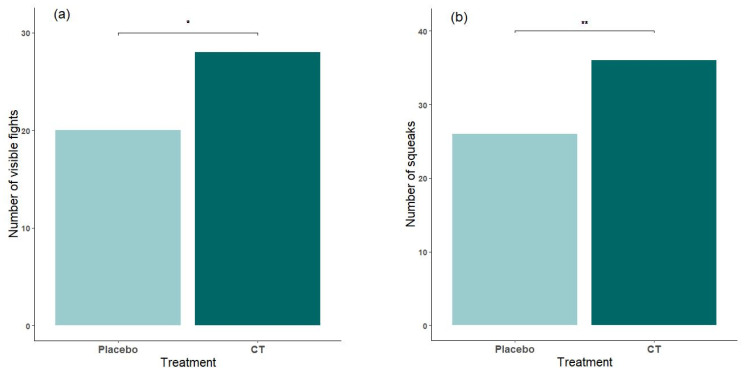
Effect of treatment on visible fights (**a**) and squeaks (**b**). ** 0.001 ≤ *p*-value < 0.01; * 0.01 ≤ *p*-value < 0.05.

**Figure 3:**
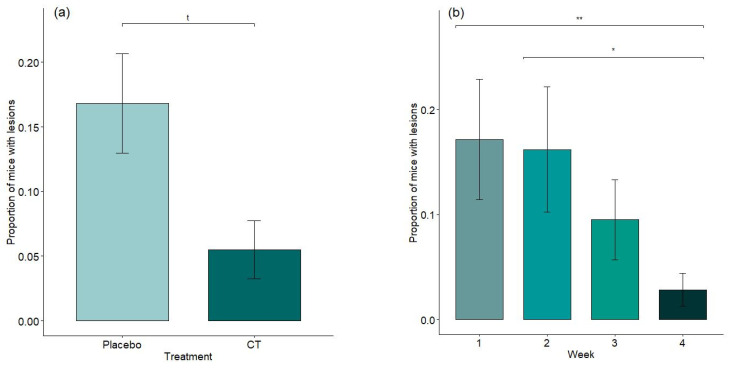
Effect of treatment on number of mice with fight lesions (**a**) and proportion of mice with lesions over the 4 weeks of habituation (all treatments) (**b**). ** 0.001 ≤ *p*-value < 0.01; * 0.01 ≤ *p*-value < 0.05; “t” statistical trend (0.05 ≤ *p*-value < 0.1). Means with standard errors (SEs) are shown.

When analyzing data from mice of all ages versus the effect of treatment, there were significantly more visible fights (*p* = 0.0249) and squeaks (*p* = 0.0048) in mice exposed to semiochemicals compared with placebo animals (Figure 2a,b). Interestingly, though, these same mice showed a tendency to have fewer lesions (*p* = 0.0697; MEAN ± SD A (Placebo): 0.17 ± 0.25; B (CT): 0.06 ± 0.14, see Figure 3a). The number of lesions also decreased during the 4 weeks of habituation time (*p* = 0.0083) (Figure 3b). All other analyzed parameters were non-significant or non-remarkable for the current analysis (see Supplementary Material, Supplementary File S1, Supplementary Table S1).

### 3.2. Effect of Treatment on Regrouping

**Figure 4:**
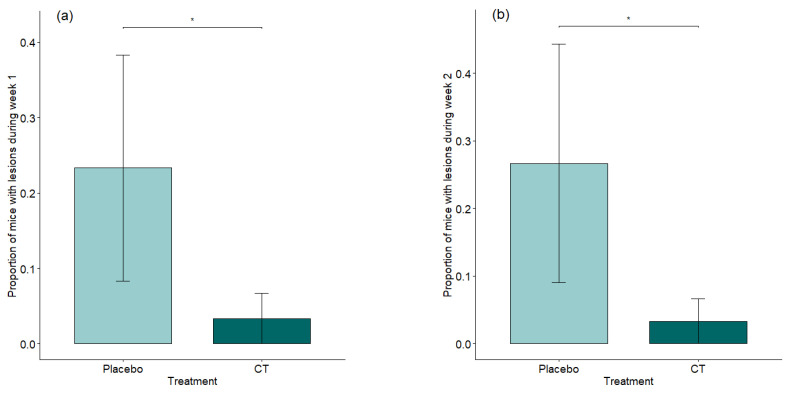
(**a**) (Week 1) and (**b**) (Week 2): Effect of treatment on number of mice with lesions in the first and second week post regrouping. * 0.01 ≤ *p*-value < 0.05. Means with standard errors (SEs) are shown.

Animals purchased at three months of age (resident population, *n* = 56) were delivered singly housed, an unplanned condition resulting from a logistical deviation. Animals were thus grouped upon arrival at IRSEA, mixing adult males to achieve standardized housing densities. The effect of unfamiliar male mixing was not initially considered as an independent factor in the study design, but was identified during a secondary analysis, following the recognition of its potential influence on aggressive behavior and treatment response. This subgroup analysis was performed post hoc, motivated by the recognition of unfamiliar male mixing as a relevant behavioral condition not accounted for in the original design. Interestingly, when we extrapolate data referring to these animals, the protective effect of treatment against the escalation of violence and fight wounds count is clear in the acute phase of mixing (see Figure 4a,b and Supplementary Material Supplementary File S1, Supplementary Table S2). The proportion of mice showing lesions was significantly lower in the treated group in the first week after mixing (*p* = 0.0215, MEAN ± SD A (Placebo): 0.23 ± 0.37; B (CT): 0.03 ± 0.08) as well as in the second (*p* = 0.0329, MEAN ± SD A (Placebo): 0.27 ± 0.43; B (CT): 0.03 ± 0.08), becoming a trend by the third week (*p* = 0.0911) and stabilizing by the fourth week (*p* = 0.2322).

### 3.3. EPM and RI Test

**Figure 5:**
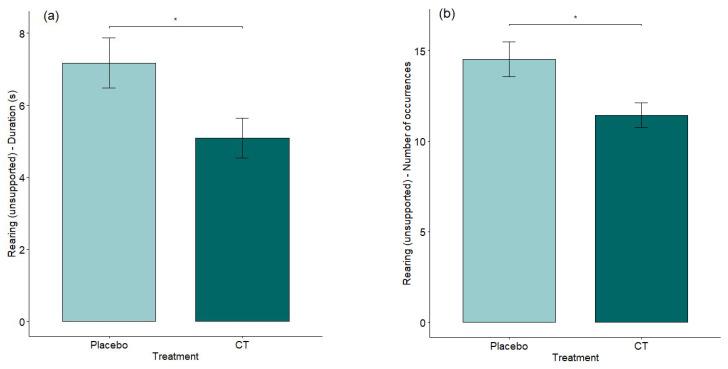
(**a**) (Duration) and (**b**) (Occurrence): Effect of treatment on unsupported rearing duration and occurrence. * 0.01≤ *p*-value < 0.05RI Test. Means with standard errors (SEs) are shown.

The effect of treatment was significant for unsupported rearing duration and occurrence during EPM analysis. We observed fewer unsupported rearing events in mice treated with semiochemicals than with a placebo (duration: *p* = 0.0284, MEAN ± SD A (Placebo): 7.17 ± 4.81 s; B (CT): 5.09 ± 3.82 s and occurrence: *p* = 0.0187, MEAN ± SD A (Placebo): 14.53 ± 6.63; B (CT): 11.44 ± 4.68, see Figure 5a,b). All other analyzed parameters were non-significant (see Supplementary Material, Supplementary File S1, Supplementary Table S3).

**Figure 6:**
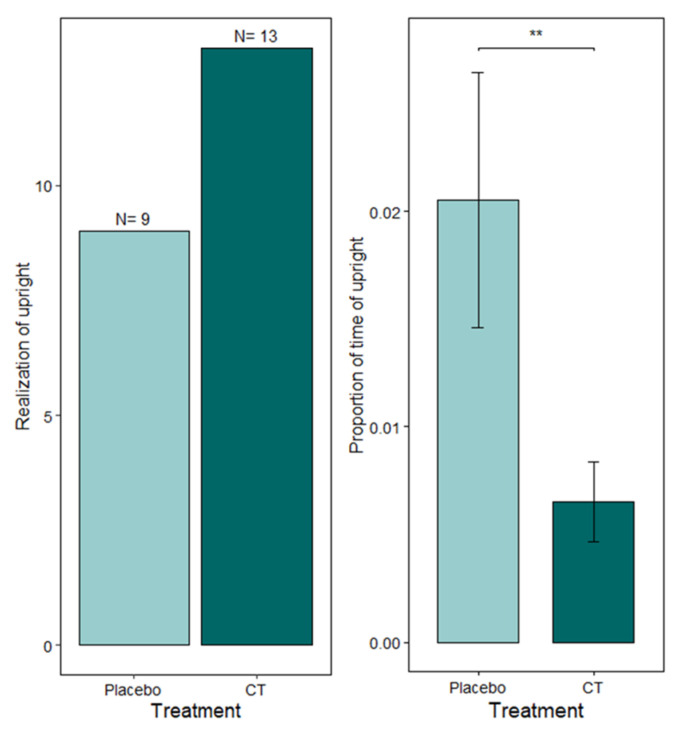
Effect of treatment on upright posture duration. ** 0.001 ≤ *p*-value < 0.01. (**Left**): binary part of the hurdle model (number of individuals with a non-zero upright posture duration); (**Right**): positive part of the hurdle model (proportion of time spent upright among individuals with non-zero duration). Means with standard errors (SEs) are shown.

A single parameter in the category of Intimidation emerged significantly for the Resident–Intruder test, namely the upright posture duration (see Figure 6). The upright postures of mice exposed to the Common Trunk of the appeasing pheromone lasted less time than those of placebo mice (*p* = 0.0031, MEAN ± SD A (Placebo): 0.02 ± 0.02 B (CT): 0.01 ± 0.01), despite a very similar occurrence, as evidenced by the average number of occurrences (*p* = 0.8274, MEAN ± SD A (Placebo): 0.01 ± 0.01 and B (CT): 0.00 ± 0.01). All other analyzed parameters were non-significant (see Supplementary Material, Supplementary File S1, Supplementary Table S4).

### 3.4. Hematobiochemistry

**Figure 7:**
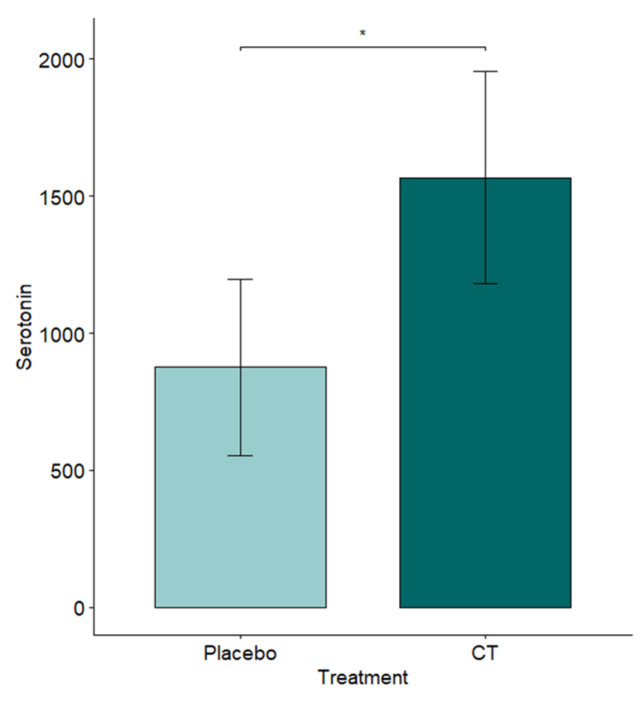
Effect of treatment on Serotonin plasma levels, ng/mL. * 0.01 ≤ *p*-value < 0.05. Means with standard errors (SEs) are shown.

Blood samples were taken from resident animals at the end of the Resident–Intruder test. Mice exposed to semiochemicals had higher levels of Serotonin (*p* = 0.0295, MEAN ± SD A (Placebo): 875.41 ± 1507.16 ng/mL; B (CT): 1566.71 ± 1638.82 ng/mL, see Figure 7). Differences between the two groups for Corticosterone and Testosterone were non-significant (see Supplementary Material, Supplementary File S1, Supplementary Table S5).

## 4. Discussion

While a previous preprint reported emotion-modulating effects of a blend of five common synthetic fatty acids [30], to the best of our knowledge, this study represents the first comprehensive and peer-reviewed analysis evaluating the efficacy of the Common Trunk (CT) of mammalian appeasing pheromone in laboratory mice. Several key findings emerge from our investigation.

Our analysis indicates that the EPM and the RI test provided only very limited insight into the effects of the semiochemical treatment. While a few parameters reached statistical significance, the overall informativeness of the tests was modest.

In the EPM, mice treated with CT exhibited a reduction in unsupported rearing, both in occurrence and duration, compared to the placebo group (*p* = 0.0187 and *p* = 0.0284, respectively). Unsupported rearing, defined as the animal lifting its forelimbs off the ground and extending its body upward on its hind legs [20], contrasts with supported rearing, in which the forepaws are placed against a surface. While many studies aggregate these behaviors under the general category of “rearing,” others distinguish between the two due to their differing ethological significance. Supported rearing is often associated with exploratory behavior, whereas unsupported rearing has been interpreted as an index of vigilance and responsiveness to novelty [21,31].

The reduced occurrence and duration of unsupported rearing observed in the treated mice may suggest a lower level of environmental vigilance, possibly reflecting improved adaptation to the novel context of the EPM. However, aside from this parameter, no other behavioral measures differentiated the two groups.

The EPM is a widely used model for anxiety-related behavior, especially in pharmacological studies testing the efficacy of anxiolytics such as benzodiazepines, where increased exploration of the open arms is taken as a proxy for reduced anxiety [32]. The test enables differentiation between anxiety-like behaviors, such as stretched attend posture (SAP) and head dipping [19], and behaviors merely related to stress or coping style. This distinction is critical, as anxiety—defined as a future-oriented mood state involving the anticipation of potential negative outcomes [33] differs from immediate stress responses or behavioral maladaptation. The lack of significant findings beyond unsupported rearing may indicate that the treatment did not substantially modulate anxiety per se.

In the RI test too, only one parameter showed a significant difference: upright posture duration. Mice exposed to CT maintained this posture for a shorter period than placebo-treated mice (*p* = 0.0031), despite a comparable number of occurrences (*p* = 0.8274). Upright posture is a defensive or offensive display used to keep an opponent at bay, commonly observed in various rodent species, including mice and hamsters [34,35]. It often occurs during the escalation or de-escalation phases of agonistic encounters. The shorter duration of upright posture in treated mice may suggest that they were more effective in defusing social tension or deterring aggression, requiring less sustained signaling.

Taken together, the data suggest that treatment may not directly affect anxiety-related states. Several non-mutually exclusive explanations are plausible: (1) the mice may not have been in a pathological state of anxiety to begin with, or the anxiety provoked by the test may have been similar across groups; (2) pheromone-based compounds may not target anxiety-specific behaviors but rather enhance environmental adaptability and social functioning. This interpretation aligns with previous findings on appeasing pheromones [5,7] and is supported here by the reduction in unsupported rearing and altered expression of upright posture.

On the other hand, and most notably, the analysis of clinical conditions, social group dynamics within the cage, and hematobiochemical parameters appeared to be substantially more informative. These data suggest that the Common Trunk (CT) of mammalian appeasing pheromone modulates prosocial behavior in male mice and promotes a more efficient expression of agonistic behaviors, facilitating conflict resolution by reducing escalation to physical aggression.

Mice exposed to semiochemicals treatment exhibited a significantly higher number of visible fights (*p* = 0.0249) and squeaks (*p* = 0.0048), compared to the placebo group. Interestingly, these same mice also tended to exhibit fewer skin lesions (*p* = 0.0697), indicating that although agonistic interactions may have been more frequent, their intensity was likely lower. Notably, two cases of severe injuries requiring euthanasia, two cases of animals found dead (one exhibiting external lesions on rump and flanks), and a single case of an aggressive mouse necessitating single housing were recorded—all occurred in cages of placebo mice. Events such as ‘found dead in cage’ or ‘euthanized due to severe injury’ were not included in the statistical analysis.

Squeaks are usually non-aggressive, defensive vocalizations emitted by subordinate individuals to express social stress and signal the aggressor to cease the attack when being pursued or bitten [29]. These vocalizations are a form of communication that, while not yet fully understood, may indicate enhanced social signaling during confrontations.

Although mice exposed to the CT of the pheromone engaged in more observable agonistic behaviors, they sustained fewer injuries, suggesting a shift toward more ritualized and communicative interactions. In this context, mice treated with CT may have been more capable of expressing social dominance or submission through posturing and vocal cues, thereby de-escalating potential aggression and preventing serious injury. These findings are consistent with previous observations in the swine [36,37].

The most striking findings, though, emerged from the welfare assessment of unfamiliar adult male mice mixed together—a known trigger for heightened aggression and risk of injury in laboratory mice [11,13,14,38]. In our study, the proportion of mice displaying visible lesions was significantly lower in the semiochemical-treated group during the first week post-mixing (*p* = 0.0215) and remained significantly lower in the second week (*p* = 0.0329). This difference became a trend in the third week (*p* = 0.0911) and stabilized by the fourth week (*p* = 0.2322).

Of note, with regard to the overall quantification of skin lesions, only visible ones were accounted for, and no specific post-mortem assessment, including shaving, was performed to check for the presence of superficial, minor, or non-visible lesions.

These behavioral findings were paralleled by physiological differences. Resident mice exposed to semiochemicals exhibited significantly higher plasma serotonin levels at the conclusion of the Resident–Intruder encounter (*p* = 0.0295). Blood samples were collected from the resident animals immediately following the test.

Serotonin is widely recognized for its role in modulating aggression. Generally, lower serotonin levels are associated with increased aggressive behavior, and such aggression has been linked to dysfunctions in serotonin receptor systems, particularly 5-HT1A receptors [39,40,41,42,43]. In the present study, the higher plasma serotonin levels in treated mice suggest that CT may influence serotonergic regulation pathways, contributing to a reduction in aggressive tendencies.

Given the relevance of information obtained during the clinical welfare assessment performed in the first 4 weeks of our study, we would like to introduce a problem-oriented approach to interpreting behavioral and welfare outcomes—particularly in the context of male group housing. Beyond individual statistical outputs, risk-based analysis can offer a broader and more practical perspective on the relevance of treatment effects. An aggregate evaluation of total injury occurrence (Supplementary Materials, Supplementary File S2)—including both visible lesions and scars—across treatment groups reveals a clear difference. Out of 209 observations, Group A (placebo) recorded a total of 78 wounds (37.3% incidence), whereas Group B (treated with the CT of appeasing pheromones) showed only 50 wounds out of 215 observations (23.3% incidence). This corresponds to an absolute risk reduction (ARR) of 14 percentage points, meaning that for every 100 mice treated, 14 injuries were prevented in the treated group. The relative risk (RR) of 0.6247 represents a 37.53% reduction in injury risk for Group B compared to Group A.

Although these differences were not statistically significant in the main inferential model, the aggregate data provide further compelling evidence for a potential protective effect of the treatment. In this sense, a risk analysis approach can complement traditional hypothesis testing by offering an applied view of the treatment’s impact on the population level, particularly relevant for animal welfare outcomes in laboratory housing.

Temporal analysis of injury risk further refines this interpretation (Supplementary Materials, Supplementary File S2). In Week 1, mice in Group A experienced an injury risk of 27.3%, compared to 12.5% in Group B—yielding an ARR of 14.8 percentage points and a relative risk reduction (RRR) of 54.2%. The difference in risk progressively reduces with time, reaching an absolute risk reduction of 6.8 points, and a relative reduction of 16.7% by week 4. These data may suggest that semiochemicals treatment seems to have a pronounced early protective effect, possibly linked to reduced aggression during the critical social reorganization phase post-mixing, as already demonstrated with other species [7,44,45].

These findings support the value of integrating risk-based metrics into welfare assessment frameworks, reinforcing the important role of semiochemicals not only in modulating individual behavior but also in shaping group-level outcomes. Ultimately, considering both statistical significance and impact on daily animal management can enhance the readability of behavioral interventions in animal welfare science.

To conclude, some limitations of the present study should be acknowledged. As differences in mouse behavioral patterns have been documented between animals housed in open cages versus individually ventilated cages (IVCs) [46,47], future assessments should include evaluations under IVCs conditions. However, it is important to note that the semiochemicals used in this study cannot be autoclaved or irradiated before their introduction into an animal facility. This presents a challenge for research and development, as well as for the validation of our methods, particularly in high-hygiene-level facilities, where such sterilization protocols are standard.

Another limitation is the overlap between the room and treatment factors, as each treatment was applied in a single room. This overlap made it statistically infeasible and inappropriate to include room as a random effect in the model, as there was no independent variability between rooms that could be separated from the treatment effect. A potential mitigation could have been a design where rooms were alternated in use over time and smaller, multiple cohorts were used, which would have better isolated the treatment effect from any potential room-related factors. However, repeating the analysis across different months or seasons would have introduced additional complexities that might have also biased experimental read-outs [48,49]. Given the exploratory nature of the study, and the secured mitigation strategies, including the standardization of workflows and human presence across rooms that were served by a dedicated service corridor, and identity in the structure, dimensions, and settings of the rooms, we focused on obtaining preliminary insights into the treatment effects by running both groups at the very same time. Future studies could certainly benefit from a design that separates rooms, or more broadly, housing type, and treatment effects more clearly.

As a final consideration, only outbred, male subjects of a specific age range were included in the present study, as the primary aim was to conduct a preliminary evaluation of the CT’s efficacy in a representative model of agonistic behavior with potential practical implications for managing male mice aggression and violence [12,50,51,52]. Further validation phases will have to consider females as well, to better understand the impact of pheromone application on the peculiar aspect of female aggressivity [53,54], as well as male mice at different life stages and ages [55], or both sexes of other strains, known to show particularly acute responses to social stress and aggressive behavior [56,57]. The same principle is valid for mouse models at different levels of hygiene conditions, subsequently showing different microbiomes, which are known to influence both anxiety-like behavior and aggression-related disorders [58,59].

## 5. Conclusions

Altogether, our findings support the notion that semiochemicals treatment has a regulatory effect on social dynamics and aggression management in male mice. Beyond its ethological implications, this treatment may offer a practical solution to common management challenges in laboratory animal facilities, including the prevention of male aggression, reduction in fight-related injuries, and improved compatibility in scenarios involving late pooling or the single-housing of males. As such, the use of the CT of appeasing pheromones may provide a promising avenue for improving welfare and space utilization in modern laboratory animal facilities and may contribute to future refinement strategies. Future lines of research should focus on broader validation of this study to better understand the applicability of pheromones with regard to different types of housing and caging systems and subsequent hygiene levels of housing, sex- and strain-specific effects, and specific challenging phases of the life of the laboratory mouse, including weaning, aging or husbandry-related activities like transport, habituation to handling, and restraining or reaction to cage-change. The compatibility of pheromones application with experimental models and specific research goals should also be carefully considered, to exclude or identify and mitigate potential bias factors.

## Figures and Tables

**Table 1:** Ethogram for mouse behavior in the Elevated Plus Maze (EPM).

Category	Behavior	Measured As	Description/Notes
**Space use**	Entries in maze areas	Occurrence	Number of entries into Open arms (O), Closed arms (C), and Platform (P); counted when hind paws cross the zone.
	Time in maze areas	Duration (s)	Time spent in Open arms (O), Closed arms (C), and Platform (P).
	Latency to enter the open arm	Latency (s)	Time before first entry into an open arm; 600 s noted if never entered.
**Stress indicators**	Stretched attend posture, unprotected and protected	Occurrence and duration	Investigatory posture with head extended. uSAP = in open arms, pSAP = in closed arms/platform.
	Head dipping	Occurrence	Mouse dips its head over the maze edges. uDip = in open arms, pDip = in closed arms/platform.
	Rearing	Occurrence and duration	Mouse stands on hindlegs. sR = supported by wall, uR = unsupported.
	Grooming	Occurrence and duration	Mouse licks, scratches, or rubs fur, often in a sitting position and a mix of grooming actions.

**Table 2:** Ethogram for mouse behavior in the Resident–Intruder (RI) test.

Category	Behavior	Measured As	Description/Notes
**Social investigation**	Social investigation	Occurrence and duration	Sniffing/touching the opponent’s body, including the anogenital area.
**Agonistic behavior**	Attack	Binary, latency, occurrence and duration	Latency to first attack (300 s if absent); forward motion with contact.
	Under attack	Occurrence and duration	The resident being attacked by the intruder.
	Offensive upright posture	Occurrence and duration	Stands on hindlimbs, pushes with forepaws, head pulled back
	Mounting	Occurrence and duration	Forelegs on opponent’s body; may include pelvic thrusting
	Threat	Occurrence	Lunging without contact or sideways rotation with piloerection
	Tail rattling	Occurrence	Rapid tail vibration, stiff posture; high arousal.
**Vocalizations**	Squeaking	Occurrence	High-pitched vocalizations; may indicate submission.
**Endpoint**	Blood (lesion presence)	Binary	Presence of bleeding; test endpoint.

## Data Availability

Extended data analyses available as Supplementary Materials. Raw data available from corresponding authors, safeguarded disclosure agreements where necessary.

## Supplementary Materials

The following supporting information can be downloaded at https://www.mdpi.com/article/10.3390/ani15223278/s1, Supplementary File S1: Extensive Results; Supplementary File S2: Risk Analysis.
