# Supplementary material for: Environmental Exposure to the Common Trunk of Mammalian Appeasing Pheromone Modulates Social Behavior and Reduces Fight Wounds in Male Mice
Source: Animals (Basel). 2025 Nov 13;15(22):3278. doi: 10.3390/ani15223278 (PMC12649689; doi:10.3390/ani15223278)
Supplement: Supplementary file 1 [file animals-15-03278-s001.zip › Animals_Supplementary material_File S1.pdf]

animals

MDPI

Article

Environmental Exposure to the Common Trunk of Mammalian Appeasing Pheromone Modulates Social Behavior and Reduces Fight Wounds in Male Mice

Sara Fuochi [1,*], Cecile Bienboire-Frosini [2], Estelle Descout [3], Miriam Marcet-Rius [1], Patrick Pageat [4] and Alessandro Cozzi [1,4,*]

1 Department of Ethics, Legislation & Animal Welfare, Research Institute in Semiochemistry and Applied Ethology (IRSEA), 84400 Apt, France
2 Department of Molecular Biology and Chemical Communication, Research Institute in Semiochemistry and Applied Ethology (IRSEA), 84400 Apt, France
3 Statistics and Data Management Service, Research Institute in Semiochemistry and Applied Ethology (ISEA), 84400 Apt, France
4 Research and Education Board, Research Institute in Semiochemistry and Applied Ethology (IRSEA), 84400 Apt, France
* Correspondence: s.fuochi@irsea-institute.com (S.F.); a.cozzi@irsea-institute.com (A.C.)

File S1 – Supplementary Material: Extensive Results

Supplementary Table S1: Welfare Indicators – whole population, A (Placebo): 52 mice; B (CT): 50 mice

**: Number and percentage concerning the modality 1 (binary variables).*

Welfare Indicators – Treatment Effect

Variable	*Descriptive statistics*		*Model used*	*DF*	*Chisq*	*p-value*
Alopecia mice	N (%) *	A : 8 (18.2)<br>B : 6 (15.0)	Binary	1	0.0003	0.9863
Number of mice with lesions	*MEAN ± SD*	*A : 0.17 ± 0.25*<br>*B : 0.06 ± 0.14*	*Binomial*	*1*	*3.2899*	*0.0697*
Number of mice with scars	MEAN ± SD	A : 0.21 ± 0.27<br>B : 0.18 ± 0.25	Poisson	1	0.2918	0.5891
Stooped posture mice	*N (%) **	*A : 8 (18.2)*<br>*B : 6 (15.0)*	*Binary*	*1*	*0.1523*	*0.6963*
Jerky gait mice	N (%) *	A : 5 (11.4)<br>B : 9 (22.5)	*Binary*	1	1.8105	0.1785
Hypervigilance mice	*N (%) **	*A : 2 (4.5)*<br>*B : 3 (7.5)*	*Binary*	*1*	*0.3204*	*0.5714*
Number of stereotyping mice	MEAN ± SD	A : 0.08 ± 0.16<br>B : 0.11 ± 0.15	Poisson	1	0.3806	0.5373
Visible fights	*N (%) **	*A : 20 (45.5)*<br>*B : 28 (70.0)*	*Binary*	*1*	*5.0311*	***0.0249***
Squeaks	N (%) *	A : 26 (59.1)<br>B : 36 (90.0)	Binary	1	7.9375	**0.0048**

Blood traces	*N (%) **	*A : 12 (27.3)*<br>*B : 11 (27.5)*	*Binary*	*1*	*0.0001*	*0.9934*
Number of correct responses	MEAN ± SD	A : 0.87 ± 0.16<br>B : 0.82 ± 0.15	Binomial	1	1.9870	0.1587
Number of intermediate responses	*MEAN ± SD*	*A : 0.09 ± 0.15*<br>*B : 0.12 ± 0.13*	*Poisson*	*1*	*0.5955*	*0.4403*
Number of bad responses	N (%) *	A : 7 (15.9)<br>B : 13 (32.5)	Binary	1	1.8793	0.1704

Supplementary Table S2: Effect of Treatment on Regrouped Mice: A (Placebo): 27 mice; B (CT): 28 mice

Variable	Type	Effect	Descriptive statistics (MEAN ± SD)	Model used	Results		
					DF	LR Chisq	p-value
Number of mice with lesions (3-month-old mice)	Discrete	Treatment – week 1	A : 0.23 ± 0.37<br>B : 0.03 ± 0.08	Poisson regression	1	5.2829	**0.0215**
		Treatment – week 2	A : 0.27 ± 0.43<br>B : 0.03 ± 0.08		1	4.5515	**0.0329**
		Treatment – week 3	A : 0.07 ± 0.16<br>B : 0.00 ± 0.00		1	2.8551	0.0911
		Treatment – week 4	A : 0.03 ± 0.08<br>B : 0.00 ± 0.00		1	1.4275	0.2322

*Supplementary Table S3: EPM summary results***:** A (Placebo): 47 mice; B (CT): 48 mice

**: Descriptive statistics were calculated on data where the behavior was performed.*

EPM - TREATMENT EFFECT

Variable	Descriptive statistics (MEAN ± SD)	Model used	DF	Chisq (Z for hurdle)	p-value
Number of entries in open arms	A : 8.55 ± 5.97<br>B : 9.27 ± 6.22	Negative Binomial	1	0.3698	0.5431
Number of entries in closed arms	A : 19.04 ± 4.75<br>B : 19.71 ± 4.44	Poisson	1	0.6171	0.4321
Number of entries in central platform	A : 27.55 ± 8.06<br>B : 28.92 ± 7.18	Poisson	1	1.6857	0.1942
Time spent in open arms	A : 104.34 ± 65.51<br>B : 111.93 ± 64.50	GLMM	1	0.3316	0.5647
Time spent in closed arms	A : 323.74 ± 86.72	GLMM	1	0.1448	0.7036

	B : 330.95 ± 95.71				
Time spent in central platform	A : 166.26 ± 67.50<br>B : 149.39 ± 58.74	GLMM	1	0.8079	0.3688
*Latency to enter in an open arm **	A : 38.24 ± 40.37<br>B : 73.36 ± 133.31	Cox	1	1.5693	0.2103
Stretched Attended Posture (unprotected)	A : 25.33 ± 18.95<br>B : 27.86 ± 19.86	GLMM	1	0.3398	0.5599
	A : 7.94 ± 4.92<br>B : 9.02 ± 5.92	Negative Binomial	1	0.8366	0.3604
Stretched Attended Posture (protected)	A : 135.94 ± 71.99<br>B : 142.44 ± 74.95	GLMM	1	0.1028	0.7485
	A : 21.09 ± 8.03<br>B : 20.44 ± 7.18	Negative Binomial	1	0.1863	0.6661
Stretched Attended Posture (total)	A : 161.26 ± 75.73<br>B : 167.50 ± 75.50	GLMM	1	0.0657	0.7978
	A : 29.02 ± 10.02<br>B : 29.46 ± 10.12	Negative Binomial	1	0.0228	0.8801
Head Dipping (unprotected)	A : 33.45 ± 20.68<br>B : 35.63 ± 20.52	Negative Binomial	1	0.1755	0.6753
Head Dipping (protected)	A : 48.15 ± 15.32<br>B : 48.38 ± 12.37	Negative Binomial	1	0.0220	0.8820
Head Dipping (total)	A : 81.60 ± 20.97<br>B : 84.00 ± 23.86	Negative Binomial	1	0.2687	0.6042
Rearing (supported)	A : 87.15 ± 31.67<br>B : 79.39 ± 26.08	GLMM	1	1.5546	0.2125
	A : 62.28 ± 17.79<br>B : 59.54 ± 15.91	Negative Binomial	1	0.5720	0.4495
Rearing (unsupported)	A : 7.17 ± 4.81<br>B : 5.09 ± 3.82	GLMM	1	4.8010	**0.0284**
	A : 14.53 ± 6.63<br>B : 11.44 ± 4.68	Poisson	1	5.5287	**0.0187**
Rearing (total)	A : 94.33 ± 32.08<br>B : 84.49 ± 26.32	GLMM	1	2.0518	0.1520
	A : 76.81 ± 19.77<br>B : 70.98 ± 16.95	Negative Binomial	1	2.2821	0.1309
Grooming	A : 22.98 ± 19.96<br>B : 12.77 ± 8.43	Hurdle	X	Zero part: -1.3451<br>Duration: -1.1661	Zero part: 0.1786<br>Duration: 0.2436
	A : 1.17 ± 2.30<br>B : 1.04 ± 1.73	Negative Binomial	1	0.0436	0.8346

Supplementary Table S4: RI Test - Treatment Effect: A (Placebo): 23 pairs; B (CT): 25 pairs

**: Number and percentage concerning the modality 1 (binary variables).*

***: Descriptive statistics were calculated on data where the behavior was performed.*

Variable	Type	Descriptive statistics		Model used	DF	Chisq (Z for hurdle)	p-value
Attack	Binary	N (%) *	A : 14 (60.9)<br>B : 16 (64.0)	Binomial	1	0.0501	0.8230
First attack **	Latency	MEAN ± SD	A : 139.19 ± 87.53<br>B : 127.04 ± 91.69	Cox	1	0.1063	0.7444
SOCIAL INVESTIGATION							
Social exploration	Continue (duration)	MEAN ± SD	A : 0.20 ± 0.10<br>B : 0.18 ± 0.08	GLMM	1	0.5549	0.4563
	Discrete (occurrence)	MEAN ± SD	A : 0.09 ± 0.03<br>B : 0.08 ± 0.03	Negative Binomial	1	2.3819	0.1227
Anogenital sniffing	Continue (duration)	MEAN ± SD	A : 0.19 ± 0.09<br>B : 0.18 ± 0.09	GLMM	1	0.2970	0.5858
	Discrete (occurrence)	MEAN ± SD	A : 0.07 ± 0.03<br>B : 0.06 ± 0.03	Negative Binomial	1	0.4208	0.5165
Total social investigation	Continue (duration)	MEAN ± SD	A : 0.39 ± 0.16<br>B : 0.36 ± 0.14	GLMM	1	0.6249	0.4292
	Discrete (occurrence)	MEAN ± SD	A : 0.15 ± 0.05<br>B : 0.14 ± 0.05	Negative Binomial	1	1.3827	0.2396
AGONISTIC BEHAVIOURS							
Aggressivity							
Attack	Continue (duration) **	MEAN ± SD	A : 0.02 ± 0.02<br>B : 0.02 ± 0.02	Hurdle	X	Zero part: -1.4338<br>Duration: 0.2637	Zero part: 0.1516<br>Duration: 0.7920
	Discrete (occurrence)	MEAN ± SD	A : 0.01 ± 0.01<br>B : 0.01 ± 0.01	Negative Binomial	1	0.0710	0.7898
Under attack	Continue (duration) **	MEAN ± SD	A : 0.03 ± 0.02<br>B : 0.02 ± 0.02	Hurdle	X	Zero part: 0.1668<br>Duration: -1.0506	Zero part: 0.8675<br>Duration: 0.2934
	Discrete (occurrence)	MEAN ± SD	A : 0.005 ± 0.010<br>B : 0.002 ± 0.006	Negative Binomial	1	0.4629	0.4963
Total aggressivity	Continue (duration)	MEAN ± SD	A : 0.02 ± 0.02<br>B : 0.02 ± 0.02	GLMM	1	0.8172	0.3660
	Discrete (occurrence)	MEAN ± SD	A : 0.01 ± 0.02<br>B : 0.01 ± 0.01	Negative Binomial	1	0.0134	0.9078
Intimidation							
Upright	Continue (duration) **	MEAN ± SD	A : 0.02 ± 0.02<br>B : 0.01 ± 0.01	Hurdle	X	Zero part: -0.8914<br>Duration: -2.9536	Zero part: 0.3727<br>Duration: 0.0031
	Discrete (occurrence)	MEAN ± SD	A : 0.01 ± 0.01<br>B : 0.00 ± 0.01	Negative Binomial	1	0.0476	0.8274

Mounting	Continue (duration) **	MEAN ± SD	A : 0.01 ± 0.01<br>B : 0.01 ± 0.01	Hurdle	X	Zero part: -0.0881<br>Duration: 1.3989	Zero part: 0.9298<br>Duration: 0.1619
	Discrete (occurrence)	MEAN ± SD	A : 0.002 ± 0.005<br>B : 0.004 ± 0.008	Negative Binomial	1	0.8775	0.3489
Threat	Discrete (occurrence)	MEAN ± SD	A : 0.003 ± 0.007<br>B : 0.005 ± 0.006	Negative Binomial	1	1.5505	0.2131
Tail rattling	Discrete (occurrence)	MEAN ± SD	A : 0.01 ± 0.01<br>B : 0.01 ± 0.02	Negative Binomial	1	1.6957	0.1929
Total intimidation	Continue (duration) **	MEAN ± SD	A : 0.02 ± 0.02<br>B : 0.01 ± 0.01	Hurdle	X	Zero part: -0.8182<br>Duration: -1.3499	Zero part: 0.4133<br>Duration: 0.1770
	Discrete (occurrence)	MEAN ± SD	A : 0.02 ± 0.02<br>B : 0.03 ± 0.03	Negative Binomial	1	1.2219	0.2690
Total agonistic behaviours	Continue (duration)	MEAN ± SD	A : 0.03 ± 0.04<br>B : 0.03 ± 0.03	GLMM	1	0.0149	0.9028
	Discrete (occurrence)	MEAN ± SD	A : 0.03 ± 0.04<br>B : 0.04 ± 0.04	Negative Binomial	1	0.4450	0.5047
VOCALISATION							
Squeak	Discrete (occurrence)	MEAN ± SD	A : 0.01 ± 0.03<br>B : 0.02 ± 0.04	Negative Binomial	1	0.0881	0.7666
LIMIT POINT							
Blood	Binary	N (%) *	A : 3 (13.0)<br>B : 2 (8.0)	Binomial	1	0.3214	0.5708

Supplementary Table S5: Hematobiochemical Results **-** A (Placebo): 23 mice; B (CT): 25 mice

HEMATOBIOCHEMISTRY - TREATMENT EFFECT

Variable	Type	Descriptive statistics		Test used	DF	Test statistic	p-value
Serotonin	Continue	MEAN ± SD	A : 875.41 ± 1507.16<br>B : 1566.71 ± 1638.82	Wilcoxon	X	W = 118.0000	**0.0295**
Corticosterone	Continue	MEAN ± SD	A : 107.51 ± 49.08<br>B : 118.63 ± 56.21	Student	46	t = -0.7278	0.4704
Testosterone	Continue	MEAN ± SD	A : 5606.84 ± 6919.39<br>B : 8451.72 ± 10077.86	Wilcoxon	X	W = 241.0000	0.4787
