# Supplementary material for: Environmental Exposure to the Common Trunk of Mammalian Appeasing Pheromone Modulates Social Behavior and Reduces Fight Wounds in Male Mice
Source: Animals (Basel). 2025 Nov 13;15(22):3278. doi: 10.3390/ani15223278 (PMC12649689; doi:10.3390/ani15223278)
Supplement: Supplementary file 1 [file animals-15-03278-s001.zip › Animals_Supplementary material_File S2.pdf]

animals

Article

Environmental Exposure to the Common Trunk of Mammalian Appeasing Pheromone Modulates Social Behavior and Reduces Fight Wounds in Male Mice

Sara Fuochi [1,*], Cecile Bienboire-Frosini [2], Estelle Descout [3], Miriam Marcet-Rius [1], Patrick Pageat [4] and Alessandro Cozzi [1,4,*]

[1] Department of Ethics, Legislation & Animal Welfare, Research Institute in Semiochemistry and Applied Ethology (IRSEA), 84400 Apt, France
[2] Department of Molecular Biology and Chemical Communication, Research Institute in Semiochemistry and Applied Ethology (IRSEA), 84400 Apt, France
[3] Statistics and Data Management Service, Research Institute in Semiochemistry and Applied Ethology (ISEA), 84400 Apt, France
[4] Research and Education Board, Research Institute in Semiochemistry and Applied Ethology (IRSEA), 84400 Apt, France
* Correspondence: s.fuochi@irsea-institute.com (S.F.); a.cozzi@irsea-institute.com (A.C.)

File S2 – Supplementary Material: Risk Analysis

Analysis of risk of injury (lesions and scars) depending on treatment: A (Placebo): 55 mice; B (CT): 56 mice

Over the four-week period

Summarized data: The risk (incidence) corresponds to the percentage of injured mice in each group.

Treatment	Total injuries	Total observations	Risk (incidence)
**A**	78	209	37.3%
**B**	50	215	23.3%

Risk comparison: A (Placebo); B (CT)

Two indicators were calculated:

Indicator	Formula	Interpretation	Results
**Risk difference (ARR)**	Risk A – Risk B	Absolute risk reduction (in % points)	14 percentage points
**Relative Risk (RR)**	Risk B / Risk A	How many times more or less risky is treatment B than treatment A?	0.6247

According to the absolute risk difference (ARR), treatment B prevented 14 cases of injury per 100 mice treated, compared with treatment A.

According to relative risk (RR), mice with treatment B were 37.53% less likely to be injured than mice with treatment A (1-0.6247). Treatment B therefore reduced the risk of injury by almost 40% compared with treatment A.

Week by week

Summarized data: The risk (incidence of lesions and scars:) corresponds to the percentage of injured mice in each group:

Week	Treatment	Total injuries	Total observations	Risk (incidence)
1	A	15	55	27.3%
	B	7	56	12.5%
2	A	21	53	39.6%
	B	12	56	21.4%
3	A	22	52	42.3%
	B	14	53	26.4%
4	A	20	49	40.8%
	B	17	50	34.0%

Week	Risk – treatment A	Risk – treatment B	ARR (% points)	RR	Relative reduction (1-RR)
**1**	27.3%	12.5%	14.8%	0.458	0.542
**2**	39.6%	21.4%	18.2%	0.541	0.459
**3**	42.3%	26.4%	15.9%	0.624	0.376
**4**	40.8%	34.0%	6.8%	0.833	0.167

Between weeks 1 and 4, a reduction in treatment efficacy was observed. Indeed, in week 1, group A had an injury rate of 27.3% versus only 12.5% in group B, representing an absolute risk reduction (ARR) of 14.8 percentage points and a relative reduction of 54.2%. In contrast, by week 4, treatment A achieved 40.8% injury-free versus 34% for treatment B, for an ARR reduced to 6.8 percentage points and a relative reduction of only 16.7%.
